# Editorial: Self-Domestication and Human Evolution

**DOI:** 10.3389/fpsyg.2020.02007

**Published:** 2020-08-25

**Authors:** Antonio Benítez-Burraco, Zanna Clay, Vera Kempe

**Affiliations:** ^1^Department of Spanish, Linguistics, and Theory of Literature (Linguistics), University of Seville, Seville, Spain; ^2^Department of Psychology, Durham University, Durham, United Kingdom; ^3^Division of Psychology, School of Health and Social Sciences, University of Abertay, Dundee, United Kingdom

**Keywords:** self-domestication, human evolution, culture, language, cognition

The human self-domestication hypothesis, which traces back to Darwin himself, has experienced a recent resurgence in interest as an account for how modern human behaviors, morphology, and culture might have evolved. Although modern humans exhibit many shared features with other closely-related species, there is evidence of a distinct suite of derived physical, cognitive, and behavioral traits which are indicative of a domestication-like process. In order to understand the evolutionary path toward these distinct human traits, we need refined evolutionary models that provide mechanistic accounts for the multiple feedback loops that occur between cultural and biological evolutionary processes, whereby selection pressures for modern human traits, including language, may have affected cultural practice, which, in turn, created niches that impacted their biological evolution. With recent advances in the field, the present volume brings together an exciting range of theoretical perspectives that aspire to this goal.

The human self-domestication hypothesis builds on the finding that, compared to extant primates and extinct hominins, humans exhibit many of the distinctive morphological, behavioral, and cognitive features also observed in domesticated animals. At least in recent specimens, these include reduced skull/brain size, neotenic features, reduced sexual dimorphism, reduced reactive aggression, increased sociability, playfulness, social tolerance as well as enhanced sensibility to social and emotional cues (see Hare, 2017 for review; and Sánchez-Villagra and van Schaik, 2019 for a critical view). Although typically done in a pre-meditated way with domesticated animals, selection for more tolerant sexual and social partners (selection against aggression) has been hypothesized to have triggered a process in humans akin to domestication. Intriguingly, it has been suggested that a similar process may have also occurred in our closest ape relatives, the bonobos, who also show a similar trait of enhanced social tolerance, reduced aggression, and a suite of other neotenous traits (Hare et al., 2012). In *Homo*, features of self-domestication have been exacerbated in our recent history, reaching their peak during the Upper Paleolithic, when crucial changes in behavior, cognition and culture are thought to have occurred (Cieri et al., 2014). Selection against aggression has been argued to facilitate the creation of the special niche favoring the emergence of complex behaviors via cultural evolution. Accordingly, self-domestication has been invoked to account for key innovations in our behavior and cognition, including enhanced cooperation and complex social networks, cumulative culture, advanced technologies, and language (Hare et al., 2012; Hare, 2017; Thomas and Kirby, 2018; Benítez-Burraco and Progovac, 2020).

The main objective of this volume is to showcase some of the most recent accounts of the human self-domestication hypothesis. Such accounts require a more comprehensive characterization of humans as self-domesticates at the morphological, cognitive, or behavioral levels, as some domains are certainly underexplored. Accordingly, the contribution by Bruner and Gleeson considers the impact of self-domestication on brain-body-tool integration, i.e., the integration between brain morphology (particularly, the parietal cortex), cognitive function (specifically, visuospatial integration), and behavior (i.e., tool manufacture and use). These authors propose a feedback effect between the expansion of the parietal cortex driven by increased neural plasticity and sociability afforded by an extended juvenile period, and improved visuospatial cognition as a prerequisite for the ability to integrate tool-use with body schemas allowing humans to off-load cognition into complex cultural practices.

A second objective of the volume is to gain greater traction on what factors may have driven the emergence of domestication features in humans. Wrangham reviews proposals for potential human self-domestication mechanisms and concludes that language, specifically, “language-based conspiracies,” acted as a driver of self-domestication by contributing to reduced reactive aggression allowing groups to unite in shared intentions of punishing aggressive individuals. A central role of language in the process of human self-domestication is also proposed in contributions by Murphy and Progovac and Benítez-Burraco. Murphy links self-domestication explicitly with models of language evolution whereas Progovac and Benítez-Burraco explore the more specific role of a feedback effect between reduced reactive aggression and improved verbal behavior as its replacement in the acceleration of human self-domestication, and advance several ideas about the nature of human languages during early Prehistory.

A third objective, addressed by four papers in this issue, is to improve our understanding of the effects of human self-domestication on the evolution of complex human social-cultural practices. Kessler explores the role of self-domestication in the emergence of human healthcare behaviors, conceived of as a merger of the capacity for social care for individuals that capitalized on human offspring care propensities, and community health behaviors which evolved independently in animals. Lenfesty and Morgan explore the interaction between prestige hierarchies that manifest themselves in religious practices and social learning of prosocial behaviors that may have contributed to self-domestication. Belfer-Cohen and Hovers present archaeological evidence relating self-domestication to the emergence of social cognition that promotes within vs. between-group categorization of conspecifics and serves as a driver of cultural evolution. Finally, Barron and Hare explore the contribution of self-domestication to the evolution and maintenance of human same-sex attraction. These authors suggest that same-sex sexual behavior reinforces enhanced prosocial tendencies such as social bonding, appeasement and play that result from, and contribute to, human self-domestication.

Finally, given an ongoing degree of controversy regarding the self-domestication hypothesis, a last objective of this volume is to present some critique and alternative accounts of human evolution. Shilton et al. identify contrasts between human social evolution and that of domesticated mammals, and conclude that rather than for reduced aggression, modern human evolution may have instead being driven by selection for socially-mediated emotional control and plasticity. This places a relevant note of caution to an approach that views human evolution exclusively as the outcome of a self-domestication process and, as a consequence, may inspire greater integration of different theoretical perspectives in future research.

Overall, this volume contributes a diverse collection of papers that tackle the exciting challenge of providing new views on human evolution which will ultimately help us to form a better understanding of the nature and the origins of human cognition, behavior, and culture.

## Author Contributions

AB-B, ZC, and VK conceived and wrote the paper. All authors contributed to the article and approved the submitted version.

## Conflict of Interest

The authors declare that the research was conducted in the absence of any commercial or financial relationships that could be construed as a potential conflict of interest.
